# Low-frequency intermediate penetrance variants in the ROCK1 gene predispose to Tetralogy of Fallot

**DOI:** 10.1186/1471-2156-14-57

**Published:** 2013-06-19

**Authors:** Julian Palomino Doza, Ana Topf, Jamie Bentham, Shoumo Bhattacharya, Catherine Cosgrove, J David Brook, Javier Granados-Riveron, Frances A Bu’Lock, John O’Sullivan, A Graham Stuart, Jonathan Parsons, Caroline Relton, Judith Goodship, Deborah J Henderson, Bernard Keavney

**Affiliations:** 1 Institute of Genetic Medicine, Newcastle University, Newcastle, UK; 2 Department of Cardiovascular Medicine, Oxford University, Oxford, UK; 3 Institute of Genetics, Nottingham University, Nottingham, UK; 4 University Hospitals of Leicester NHS Trust, Leicester, UK; 5 Newcastle upon Tyne Hospitals NHS Foundation Trust, Newcastle, UK; 6 Bristol Royal Hospital for Children, Bristol, UK; 7 Leeds Teaching Hospitals NHS Trust, Leeds, UK

**Keywords:** Congenital heart disease, Tetralogy of fallot, Genetics, Planar cell polarity pathway

## Abstract

**Background:**

Epidemiological studies indicate a substantial excess familial recurrence of non-syndromic Tetralogy of Fallot (TOF), implicating genetic factors that remain largely unknown. The Rho induced kinase 1 gene (ROCK1) is a key component of the planar cell polarity signalling pathway, which plays an important role in normal cardiac development. The aim of this study was to investigate the role of genetic variation in ROCK1 on the risk of TOF.

**Results:**

ROCK1 was sequenced in a discovery cohort of 93 non-syndromic TOF probands to identify rare variants. TagSNPs were selected to capture commoner variation in ROCK1. Novel variants and TagSNPs were genotyped in a discovery cohort of 458 TOF cases and 1331 healthy controls, and positive findings were replicated in a further 209 TOF cases and 1290 healthy controls. Association between genotypes and TOF was assessed using LAMP.

A rare SNP (c.807C > T; rs56085230) discovered by sequencing was associated with TOF risk (p = 0.006) in the discovery cohort. The variant was also significantly associated with the risk of TOF in the replication cohort (p = 0.018). In the combined cohorts the odds ratio for TOF was 2.61 (95% CI 1.58-4.30); p < 0.0001. The minor allele frequency of rs56085230 in the cases was 0.02, and in the controls it was 0.007. The variant accounted for 1% of the population attributable risk (PAR) of TOF. We also found significant association with TOF for an uncommon TagSNP in ROCK1, rs288979 (OR 1.64 [95% CI 1.15-2.30]; p = 1.5x10^-5^). The minor allele frequency of rs288979 in the controls was 0.043, and the variant accounted for 11% of the PAR of TOF. These association signals were independent of each other, providing additional internal validation of our result.

**Conclusions:**

Low frequency intermediate penetrance (LFIP) variants in the ROCK1 gene predispose to the risk of TOF.

## Background

Congenital heart disease affects approximately 1% of live births and is a major source of morbidity and mortality in childhood. Tetralogy of Fallot (TOF) is the commonest cyanotic CHD, affecting approximately 3 per 10,000 newborns [1]. TOF is characterized by right ventricular outflow tract obstruction, a ventricular septal defect between the anterior and posterior limbs of the trabecular septal band, over-riding of the aorta and right ventricular hypertrophy.

Approximately 20% of TOF among live born children occurs in the setting of chromosomal conditions (notably 22q11 deletion syndrome), other multi-system malformation syndromes, or maternal factors such as teratogen exposure and diabetes. Recurrence risk studies in the remaining ~80% of “sporadic” cases indicate a significant familial predisposition, implicating genetic factors [2,3]. Very rare or private variants in candidate genes such as Nkx2.5 and Tbx1 have been shown in previous studies to account for small proportions of the population attributable risk of TOF [4,5]. Recently, association between rare copy number variants [6,7] and between commoner genetic variation and TOF has been demonstrated [8] but the associations discovered so far account for only a small proportion of the estimated heritability of the condition.

**Figure 1:**
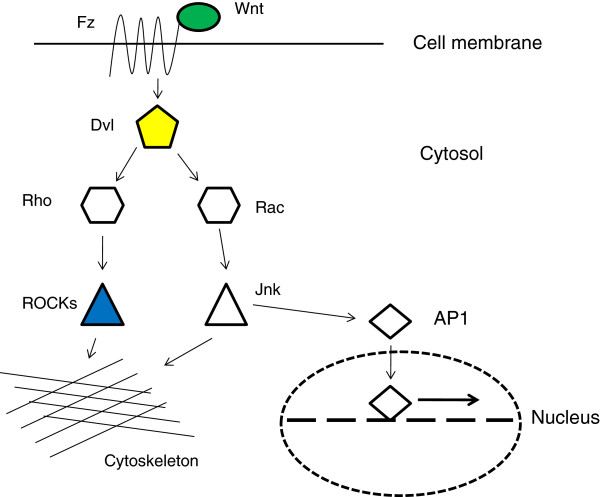
Simplified schema of the PCP pathway.

The non-canonical Wnt signalling pathway, also known as the planar cell polarity (PCP) pathway, plays a key role in cardio genesis [9,10]. The PCP pathway is principally involved in the determination of cellular polarity in the orientation perpendicular to the apical/basal plane. PCP pathway signalling is triggered by the interaction between a Wnt protein and a Frizzled (Fz) receptor, which via the protein Dishevelled, activates downstream signalling. Rho induced kinases (ROCKs) are a family of serine/threonine kinases that act as downstream effectors of PCP signalling and other signalling cascades. They phosphorylate a variety of cellular substrates and thereby influence cell polarity, adhesion and motility. A simplified schema of the principal proteins involved in PCP signalling is presented in Figure 1. ROCK1 is expressed in critical structures during the process of cardio genesis in both mouse and Xenopus embryos [11]. Inhibition of ROCKs in murine embryos induces defects in cardiac looping and septation, possibly due to effects on mesodermal cell migration [11,12]. Although the ROCK1 knockout mouse does not show a cardiac phenotype, possibly due to functional redundancy between ROCK family members in the mouse [13], phenotypic correspondence between mouse models of heart development and human disease is known to be imperfect. For example, cardiovascular malformation is not seen in EVC -/- mice which are deficient in the gene causing human Ellis van Creveld syndrome, a multisystem malformation disorder in which cardiovascular malformation is a prominent feature [14].

ROCK1 is therefore a strong candidate gene for involvement in human CHD susceptibility. We explored the involvement of both low frequency and common genetic variation in ROCK1 on the risk of TOF in a case/control study.

## Methods

### Study populations

British Caucasian patients with TOF (adults or children) were recruited from seven participating UK congenital heart disease units. Northern and Yorkshire Multicentre Ethics Committee and Central Oxford Research Ethics Committee approved the study, which was carried out following the principles of the Helsinki declaration. All patients (or their parents, if the patient was a child too young to consent him/herself) gave informed consent. The presence of a recognized genetic syndrome associated with congenital heart disease (for example DiGeorge, Noonan’s or Williams’ syndromes), or of developmental delay, were exclusion criteria. In addition to review of the clinical records, patient samples underwent screening for 22q11 deletion using a commercially available Multiplex Ligation-dependent Probe Amplification (MLPA) kit (MRC-Holland) and the sample was excluded from analysis if a deletion was confirmed. Following exclusions, 667 TOF proband samples were available for study.

Controls were British Caucasians free of self-reported CHD recruited to two previously described population-based studies [15,16]. Although controls did not undergo echocardiographic examinations or clinical assessment for CHD, misclassification due to undiagnosed TOF in any controls would have been extremely unlikely to have occurred. A total of 2715 control samples were available for study. Cases and controls were arbitrarily subdivided into test and replication cohorts.

### Rare variant discovery and replication

Screening for low frequency variants in ROCK1 was performed using Sanger sequencing in 93 TOF probands. Primers were designed to amplify exonic sequence and 100 base pairs into the intron at either end. Standard methodology was used. Optimized primer sequences, annealing temperatures and product lengths are listed in Additional file 1: Table S1. All previously undescribed variants were genotyped in the remainder of the cases and controls in the test population using SEQUENOM iPLEX assays. Assays were designed using the RealSNP web based resource. The replication cohort was genotyped for rs56085230, which achieved statistical significance at p < 0.05 in the test cohort, using the same SEQUENOM iPLEX assay. Additional file 1: Table S2 shows PCR primers, extension primers, mass and base call for the three variants in ROCK1 that were undescribed at the time of the sequencing study.

### TagSNP study of ROCK1

For the assessment of common variation in ROCK1, allele frequencies for SNPs in the first 28 exons of ROCK1 and 15Kb upstream were downloaded from the HapMap phase 2 CEU data (http://www.hapmap.org). SNPs from exons 28–31 of ROCK1 were not included since during the mutational screening a duplicated region of 19,413 bp (chr 18: 16774338..16793806) incorporating those exons was discovered to be present in all probands and control individuals. A tagging strategy with an r^2^ threshold of 0.8 and mAF of 0.05 was adopted. Using these criteria a total of 34 SNPs could be captured using 12 tagSNPs. We forced the inclusion of the previously described non-synonymous SNPs rs2271255 (Lys222Glu), rs45449301 (Ile432Val) and rs2292296 (Leu1097Phe). SNPs were also genotyped using SEQUENOM iPLEX assays. Further detail of SNPs, primers and conditions are presented in Additional file 1: Table S3.

The conformation of genotypes to Hardy-Weinberg equilibrium was checked and allele frequencies determined using PEDSTATS software [17]. Association tests were carried out using LAMP version 0.0.9 (--fastAssoc switch) [18]. To make some allowance for the risk of false-positive findings due to multiple comparisons, the results were interpreted based on the false discovery rate (FDR) using the program QVALUE [19]. We adopted an FDR of 0.05, that is, one in twenty of the associations detected at this level is anticipated to be false. Where statistically significant association was observed, we calculated the population attributable fraction for the risk genotype using Levin’s formula.

## Results

### Novel low frequency variants

Three variants undescribed at the time of the sequencing study were found in screening of the first 26 exons of ROCK1 in 93 TOF probands. The first variant was a C to T substitution in exon 7 at position 807 of the mRNA transcript (c.807C > T). The c.807C > T variant has been recently identified by the 1000 Genomes Project and assigned the identifier rs56085230. This variant causes no change to the sequence of the ROCK1 protein where a leucine is encoded at residue 268. Sequence traces for the variant are shown in Additional file 2: Figure S1. The second variant was a T to G substitution in exon 16, at position 1785 of the mRNA transcript (c.1785 T > G), which causes no change to the sequence of the ROCK1 protein where a serine is encoded at residue 595. The third variant was a C to G substitution at position 2318 of the mRNA transcript (c.2318C > G) which results in a change in amino acid at residue 773 from Threonine to Serine (p.Thr773Ser). Each of the three variants was present in one patient in the screening cohort, and no patient carried more than one variant.

The three novel variants were genotyped initially in a test cohort of 458 TOF cases and 1331 controls. We did not find significant differences in allele and genotype frequencies for c.1785 T > G and c.2318C > G. However, the minor allele of rs56085230 was significantly associated with TOF risk (p = 0.006). Genotype frequencies in the test cohort for the novel variants are shown in Table 1. In the replication cohort (consisting of 209 TOF cases and 1290 controls), rs56085230 was also significantly associated with TOF (p = 0.018). In the total population of cases and controls the odds ratio for TOF was 2.61 (95% CI 1.58-4.30; p < 0.0001) and the calculated population attributable risk was 1%. Genotype counts are shown in Table 2. The association between rs56085230 and TOF risk remained significant (p < 0.05) after correction for multiple testing using QVALUE. Analysis using ESEfinder [20] and Spliceview [21] showed that rs56085230 decreases the strength of the splicing acceptor site at the end of the exon and produces disappearance of a SrP40 binding site. Analysis of the variant using the PhastCons algorithm at the UCSC genome browser [22] showed that this nucleotide is highly conserved among mammals and vertebrates (Score = 1).

**Table 1:** Genotypes at the three previously undescribed rare variants in ROCK1 in case and control populations

	**Cases**			**Controls**			**Trend p**-**value**
**Variant**	**Homozygote major allele**	**Heterozygote**	**Homozygote minor allele**	**Homozygote major allele**	**Heterozygote**	**Homozygote minor allele**	
rs56085230	445	12	0	1283	5	0	0.006
c.1785 T > G	452	5	0	1309	10	0	0.49
p.Thr773Ser	441	17	0	1285	46	0	0.77

**Table 2:** Genotype counts for rs56085230 in the replication cohort

	**Cases**			**Controls**			**Trend p**-**value**
**Variant**	**Homozygote major allele**	**Heterozygote**	**Homozygote minor allele**	**Homozygote major allele**	**Heterozygote**	**Homozygote minor allele**	
rs56085230	195	14	0	1258	30	2	0.018

During the period this work was conducted, genome wide data on common SNPs typed on the Illumina 660 W platform became available in a proportion of cases and controls in this study. Principal components analysis of these data indicated that all carriers of the rare allele at rs56085230 clustered with the HapMap CEU population, ruling out population stratification as a cause of the association we observed. The minor allele frequency at rs56085230 was 0.02 in the cases and 0.007 in the controls; the allele frequency in controls closely agrees with that reported in the NCBI Exome Variant Server in healthy people of White European origin (0.008; http://www.evs.gs.washington.edu).

### Haplotype-tagging SNP genotyping

Genotyping was successful for at least 98% of the samples for all SNPs. The estimated genotype miscall rate was <1%. All markers were in Hardy-Weinberg equilibrium at the 5% significance level, and observed allele frequencies agreed well with HapMap data from the CEU population (Additional file 1: Table S4). Seven common haplotypes with frequencies above 1% accounted for more than 95% of common variation within the population (Additional file 1: Table S5). Genotype at the rs288979 SNP in intron 16 was significantly associated with risk of TOF (OR: 1.64 [95% CI 1.15-2.30]; p = 1.5x10^-5^; Table 3). The association remained significant at the 5% FDR level after correction for multiple testing. The minor allele frequency at rs288979 in the total population was 0.043, and the PAR for genotype at rs288979 was 11%. In silico splicing analysis of the variant showed no hypothetical effect over splicing. Analysis of the variant using the PhastCons algorithm at the UCSC genome browser showed that this nucleotide is not conserved among mammals and vertebrates (Score = 0).

**Table 3:** rs288979 Genotype counts and frequencies for probands and controls

	**A**/**A**	**A**/**G**	**G**/**G**	**MAF**	**mAF**
Probands	387	41	9	0.93249	0.06751
Controls	1658	126	4	0.96253	0.03747

**Figure 2:**
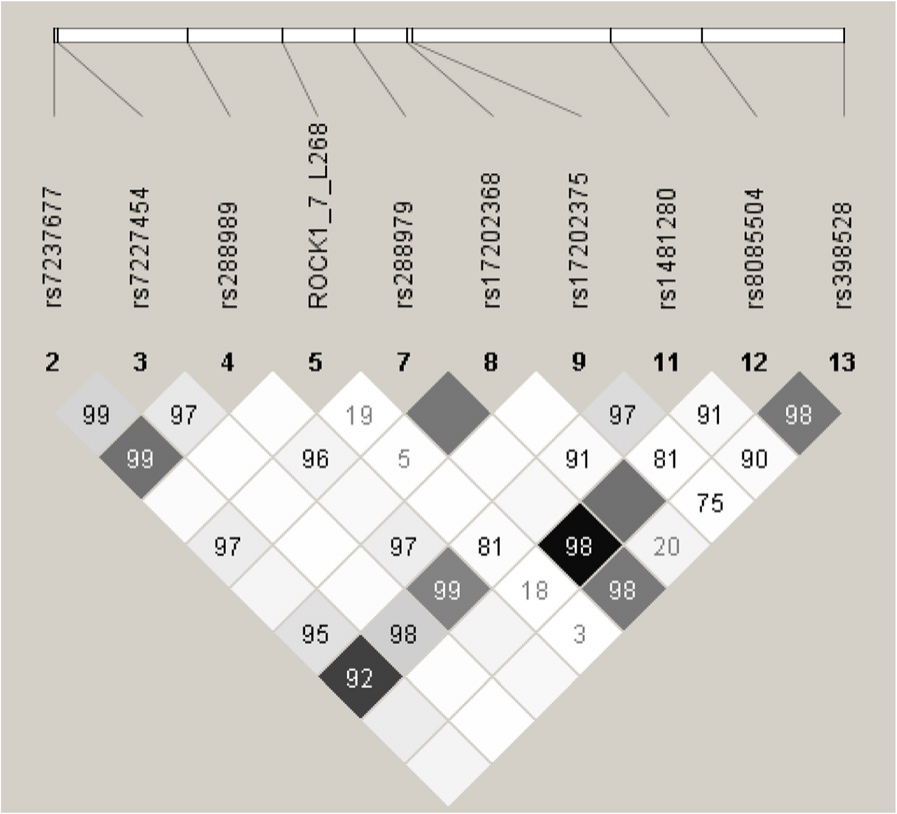
**SNPs genotyped (mAF > 0**.**001) and relationships between them.** The linkage disequilibrium relationships between SNPs are represented by Haploview triangle plots; darker small squares denote higher linkage disequilibrium (represented by r2), and the numbers in the small squares show the significant pairwise D-prime values derived from the present study.

There was no significant LD between rs288979 and rs56085230 (D′=0, r^2^ = 0; Figure 2), indicating that the association signals from these two SNPs are independent of, and therefore confirm, each other. In silico analysis of rs288979 showed no evidence of splicing changes associated with it. No other common ROCK1 SNP was associated with TOF at p < 0.05.

## Discussion

We present evidence for association between two low frequency variants in the ROCK1 gene and TOF risk. Sequencing studies detected a novel rare synonymous variant in exon 7 of ROCK1 (c.807C > T) in a highly evolutionary conserved nucleotide within the kinase motif of the protein which, according to in silico analysis, might influence splicing. During the course of this study, the variant was identified in the 1000 Genomes Project and assigned the identifier rs56085230. The variant was found to significantly increase the risk of TOF in a test cohort including 458 cases and 1331 controls. The association was replicated in 209 cases and 1290 controls, and association in the whole population remained significant after correction for multiple comparisons. The minor allele frequency of rs56085230 was approximately 0.007 in our healthy population. Since false positive results due to population stratification are a particular concern in studying rare variants, we used genome wide common SNP data to confirm that carriers of the rare allele at rs56085230 and controls were ethnically homogeneous. Additionally, TagSNP studies of ROCK1 showed association between TOF and a second, intronic variant, rs288979, whose minor allele frequency in the healthy population was 0.037. The evolutionary conservation of rs288979 was low, suggesting that it is in linkage disequilibrium with a causative variant, rather than having a direct effect on TOF risk. There was no significant linkage disequilibrium between the rs56085230 and rs288979 SNPs, thus they may be considered as providing independent replication of the contribution of the ROCK1 gene to TOF risk. To our knowledge this is the first study to systematically examine genetic variation at ROCK1 and TOF. Also, our sample is among the largest thus far reported for the genetic study of CHD; we were therefore able to carry out internal replication of the association we observed.

The finding that low frequency intermediate penetrance variants are associated with TOF, and confer moderate odds ratios, is in keeping with what might be expected from consideration of the natural history of TOF in an evolutionary context. Prior to the modern cardiac surgical era, some 80% of children born with TOF died prior to the age of ten years, suggesting that any variant that significantly increased the risk of TOF would be subject to purifying selection in the population and thus (in the absence of balancing positive selection for some other character) uncommon [23,24]. We and others have recently demonstrated association between rare genic copy number variants, which are known to be subject to strong purifying selection, and CHD risk. Some associated CNVs have similar frequencies in cases, and associated odds ratios, to the variants associated in the present study, for example 1q21.1 duplication is present in just under 1% of subjects with TOF and confers an odds ratio of around thirty [7]. The odds ratios and allele frequencies of our two associated variants are inversely related, consistent with the operation of selective pressure: whereas rs288979 has a higher mAF it confers a modest OR, and the rarer variant rs56085230 confers a correspondingly higher risk of TOF [25].

The calculated population attributable risk (PAR) for ROCK1 rs56085230 was 1% in our sample, while that for rs288979 was 11%. Notwithstanding these figures, neither variant can be considered as having an optimal combination of moderate allele frequency and high risk to carriers to be of potential use in population screening [26]. Rather, the principal utility of our result lies in the implication of ROCK1 in human outflow tract malformation and as a basis for further mechanistic studies in man. The rs56085230 variant is synonymous, and minigene splicing studies showed no evidence that it acts to affect splicing (data not shown); the rs288979 variant is intronic and of unknown function. The mechanisms of action of these variants therefore require further study.

“Low frequency intermediate penetrance” (LFIP) variants such as rs56085230 and rs288979 are presently the subject of intensive investigation as the potential source for the substantial “missing heritability” not detected by GWAS approaches in many complex diseases. However, there is relatively little evidence thus far that such variants do indeed make significant contributions to complex disease risk. The present study provides, to our knowledge, the first evidence that LFIP variants contribute to the risk of sporadic, non-syndromic congenital heart disease. Our study highlights some issues particular to the study of such LFIP variants that merit comment. First, despite our study being among the largest of CHD genetics thus reported, the p-values for association that we obtained were modest. This is an inevitable consequence of the low frequency of the associated variants. In order to achieve significance at a level typically considered acceptable in GWAS studies of commoner variants (p < 5x10^-8^), hundreds of thousands of CHD patients and controls would have been required. While this was unfeasible, our study importantly demonstrated internal replication, since two ROCK1 variants independently showed association with TOF; it is likely that such internal replication by independent SNPs in the same gene, in the same study population, will be an important feature of future LFIP studies. Second, studies of LFIP variants are inherently more susceptible to confounding by population stratification than are studies of commoner variants. We used genome-wide SNP chip data in our cases and controls to confirm that rare variant carriers clustered with the HapMap CEU population; however, only family-based studies would have the capacity to entirely remove concerns about small degrees of population stratification not detected by this approach.

Further studies will be required to determine how both rs56085230 and the intronic rs288979 SNP, which has no known function, influence TOF risk; study in the appropriate human cell type in an environment corresponding to early organogenesis will likely be challenging. The hypothetical influence of rs56085230 over splicing should be explored in the future. It remains possible that the association observed at these SNPs is due to LD with other LFIP variants that were untyped in this study; further sequencing and genotyping studies will be required to resolve this. Confirmation of the result we have obtained for TOF in a similarly large cohort of patients with other congenital heart disease diagnoses would be of interest to establish whether the influence of ROCK1 is restricted to outflow tract defects. Notwithstanding these limitations, our study provides the first evidence in man that disturbances of PCP pathway signalling plays a role in the aetiology of cardiac malformations. Study of other genes in the PCP pathway, and mouse modelling studies to further elucidate the role of ROCK1 in cardiac development would be of significant interest.

## Conclusions

We found evidence of two significant associations between low frequency variants in ROCK1, a plausible candidate gene for human cardiac malformations, and the risk of TOF. This is the first large cohort study exploring the relationship between genetic variation at the ROCK1 gene and human cardiac malformations. These results also provide among the first evidence that low frequency intermediate penetrance variants explain a significant proportion of genetic predisposition to certain complex diseases.

## Competing interests

The authors declare that they have no competing interests.

## Authors’ contributions

JP participated in the design, performed the sequencing and the Sequenom assays, participated in the statistical analysis and drafted the manuscript; AT participated in the sequencing and the Sequenom assays and also in the statistical analysis; CC, DB, JG, FB, JO, GS, JP, JB and CR recruited patients, collected the samples and participated in the design and coordination of the study; JG and DH participated in the study design, data analysis and manuscript draft; BK participated in the design, coordination and supervision of the study, data analysis and manuscript draft. All authors read and approved the final manuscript.

## Supplementary Material

Additional file 1: Table S1 ROCK 1 ROCK 1 primers, optimal annealing temperature and PCR product length. **Table S2.** PCR primers, extension primer s, masses and base call for the MassExtend genotyping experiment for the previously undescribed variants. **Table S3.** PCR primer extension primer and mass for the ROCK1 tagged SNPs Sequenom assay. **Table S4.** Counts and allele frequencies for our population and Hapmap CEU data, Hom WT: Homozygotes wild type; Hom NWT: Homozygotes not wild type; mAF: minor allele frequencies; MAF: major allele frequencies; Het Heterozygotes. **Table S5.** Common Haplotypes for ROCK1 within our research population. Genotypes are specified for the following SNPs: rs7227454, rs288989, 807 C > T, rs288979, rs17202368, rs17202375, rs1481280, rs8085504 and rs398528. Click here for file

Additional file 2: Figure S1 ROCK1 807C > T variant trace display. Upper and medium panel show patient sequence, bottom shows a normal trace. Click here for file
