# Supplementary material for: Low-frequency intermediate penetrance variants in the ROCK1 gene predispose to Tetralogy of Fallot
Source: BMC Genet. 2013 Jun 19;14:57. doi: 10.1186/1471-2156-14-57 (PMC3734041; doi:10.1186/1471-2156-14-57)
Supplement: Additional file 1: Table S1 — ROCK 1 ROCK 1 primers, optimal annealing temperature and PCR product length. Table S2. PCR primers, extension primer s, masses and base call for the MassExtend genotyping experiment for the previously undescribed variants. Table S3. PCR primer extension primer and mass for the ROCK1 tagged SNPs Sequenom assay. Table S4. Counts and allele frequencies for our population and Hapmap CEU data, Hom WT: Homozygotes wild type; Hom NWT: Homozygotes not wild type; mAF: minor allele frequencies; MAF: major allele frequencies; Het Heterozygotes. Table S5. Common Haplotypes for ROCK1 within our research population. Genotypes are specified for the following SNPs: rs7227454, rs288989, 807 C > T, rs288979, rs17202368, rs17202375, rs1481280, rs8085504 and rs398528. [file 1471-2156-14-57-S1.docx]

Supplementary table 1. ROCK1 primers, optimal annealing temperature and PCR product length

Supplementary Table 2. PCR primers, extension primers, masses and base call for the MassExtend genotyping experiment for the previously undescribed variants.

Supplementary Table 3. PCR primer extension primer and mass for the ROCK1 tagged SNPs Sequenom assay

Supplementary Table 4. Counts and allele frequencies for our population and Hapmap CEU data. Hom WT: Homozygotes wild type; Hom NWT: Homozygotes not wild type; mAF: minor allele frequencies; MAF: major allele frequencies; Het: Heterozygotes.

Supplementary Table 5. Common Haplotypes for ROCK1 within our research population. Genotypes are specified for the following SNPs: rs7227454, rs288989, 807 C>T, rs288979, rs17202368, rs17202375, rs1481280, rs8085504 and rs398528.
