# Supplementary material for: Low-frequency intermediate penetrance variants in the ROCK1 gene predispose to Tetralogy of Fallot
Source: BMC Genet. 2013 Jun 19;14:57. doi: 10.1186/1471-2156-14-57 (PMC3734041; doi:10.1186/1471-2156-14-57)
Supplement: Additional file 2: Figure S1 — ROCK1 807C > T variant trace display. Upper and medium panel show patient sequence, bottom shows a normal trace. [file 1471-2156-14-57-S2.docx]

Supplementary Figure 1. ROCK1 807C>T variant trace display. Upper and medium panel show patient sequence, bottom shows a normal trace.
